# Physical Activity Levels and Social Cognitive Theory Correlates among Adults with Crohn’s Disease: Preliminary Results from a Cross-Sectional Analysis

**DOI:** 10.3390/ijerph21040462

**Published:** 2024-04-10

**Authors:** Whitney N. Neal, Dorothy Pekmezi, Robert W. Motl

**Affiliations:** 1 Department of Health Behavior, University of Alabama at Birmingham, Birmingham, AL 35294, USA; dpekmezi@uab.edu; 2 Department of Kinesiology and Nutrition, University of Illinois at Chicago, Chicago, IL 61820, USA; robmotl@uic.edu

**Keywords:** health behavior, physical activity assessment, chronic disease, Crohn’s disease

## Abstract

There is increasing research and clinical interest in physical activity (PA) as an adjuvant therapy for improving health outcomes among persons with Crohn’s disease. To date, little is known regarding PA behavior and its cognitive and behavioral correlates in Crohn’s disease. Thus, we assessed self-reported PA and its social cognitive theory (SCT) correlates in a sample of persons with Crohn’s disease. Data on demographic and clinical characteristics, disease activity, leisure-time PA, and SCT variables were collected from 30 participants with Crohn’s disease (90% White, 60% female) through an online survey. SCT variables assessed included exercise self-efficacy, social support, outcome expectations, goal setting, and planning. Analyses involved comparing PA levels and SCT survey scores using independent sample *t*-tests and non-parametric bivariate correlations. The majority of participants were in clinical remission (60%) and over half (57%) were classified as physically active, yet the mean PA level was lower than normative values for adults. Females (*n* = 18) and participants who reported previous surgery for Crohn’s disease (*n* = 18) were almost twice as physically active as male participants and those without a history of Crohn’s disease-related surgery, respectively (*p*’s < 0.05). Overall, participants who reported greater exercise goal setting behaviors had higher levels of PA (r_s_ = 0.34; *p* < 0.05). These findings highlight lower overall levels of PA in persons with Crohn’s disease, and exercise goal setting represents a potential target of behavior change interventions for increasing PA in this population.

## 1. Introduction

Crohn’s disease (CD) is a chronic inflammatory disorder of the gastrointestinal tract with diverse physical symptoms including diarrhea, abdominal pain, and weight loss [1]. As a result of disease-specific factors, treatment, and malnutrition, many adults with CD further experience secondary health complications (e.g., joint pain, skin lesions, osteoporosis, fatigue) [2]. Ultimately, the disease process results in not only impaired health-related quality of life (HRQOL) [3], but there is also evidence suggesting that aerobic fitness, muscle function, and bone health are worse in adults with CD than the general population [4,5,6,7]. 

Because there is no known cure for CD, increasing attention is being directed towards second-line approaches for disease management and treatment. Physical activity may be a useful adjuvant therapy for individuals with CD, as it is an essential component of symptom management and rehabilitation in many chronic diseases, with documented improvements in aerobic fitness, muscle function, fatigue, and HRQOL [8]. There is increasing evidence of improvements in clinical disease activity, muscle function, cardiorespiratory fitness, and HRQOL among physically active adults with CD [9]. One US study reported that higher physical activity levels were associated with a 32% reduction in risk of relapse in adults with CD in remission [10]. Limited trials have demonstrated that physical activity is feasible, safe, and may have beneficial effects on muscle function, bone health, and psychological well-being in this population, yet most intervention studies reported limitations such as poor adherence and retention [9]. Results from a 12-month home-based resistance training program indicated a 58% adherence rate (70/120 sessions completed), with only 14 of 53 intervention group participants completing all 120 resistance training sessions [11]. Another randomized trial that evaluated the effects of a home-based resistance training and aerobic exercise intervention reported a significantly lower dropout rate in the resistance training group compared to the aerobic exercise group (13.3% vs. 47.6%, respectively) [12]. The benefits, yet low rate of participation underscore the need for formative research on designing behavior change interventions for promoting physical activity engagement in this population. 

Previous research has primarily focused on physical activity behavior in all inflammatory bowel disease (IBD) patients, including ulcerative colitis (UC) and indeterminate colitis. CD, UC, and indeterminate colitis are all chronic and recurrent conditions characterized by inflammation of the bowels, but these are distinct diseases that differ in clinical features and therapeutic responses. For example, preliminary evidence suggests that individuals with CD are at a slightly higher risk of fractures [13] and exhibit lower aerobic exercise capacity [4] compared with healthy controls and those with UC. These differences may impact physical activity engagement differently. As such, we recently reported the results of a scoping review of studies investigating physical activity specifically in adults with CD [9]. Overall, estimates of physical activity levels among persons with CD varied widely by method of physical activity measurement and geographical location. Indeed, although adults with CD generally had lower or similar PA rates compared to controls, the nine studies that examined physical activity levels used six different self-report measures to examine physical activity behavior, highlighting the need for additional research in this area [9]. There is lack of consensus among researchers regarding the best measure of physical activity, yet the Godin Leisure Time Exercise Questionnaire (GLTEQ) and International Physical Activity Questionnaire (IPAQ) were the most common validated self-report measures for examining physical activity in adults with CD [9]. The GLTEQ [14] is a simple measure of physical activity with no specified time component that provides a nuanced understanding of weekly physical activity patterns and intensity, while the IPAQ [15] offers a more focused assessment of physical activity behavior by measuring frequency and duration of time spent in walking, moderate activity, and vigorous activity over the previous week. The IPAQ further contains an item regarding sitting as a measure of sedentary behavior. We note that previous studies that used the IPAQ to assess physical activity behavior reported high rates of missing values for the duration of walking, moderate activity, and vigorous activity. As such, an alternate scoring system for the short-form version of the IPAQ was developed in a population of adults with multiple sclerosis [16]. This involves removing physical activity duration from the calculation of an overall physical activity score and results in an IPAQ metabolic equivalent outcome that is similar with the GLTEQ. 

The evidence from cross-sectional studies indicated that disease-related variables were correlated with physical activity among adults with CD (i.e., age at diagnosis [17], disease duration [18], presence of active disease [18]), but few researchers have focused on modifiable cognitive and behavioral factors associated with physical activity engagement. These modifiable variables may explain variation in physical activity levels among adults with CD and represent potential intervention targets. Indeed, there is extensive evidence demonstrating the efficacy of physical activity interventions grounded in behavior change theory among individuals with chronic health conditions [8,19]. Such studies intervene on physical activity by targeting intermediate, theory-based constructs through behavior change techniques. Social cognitive theory (SCT) is one of the most widely applied theories for understanding physical activity behavior, and posits interplay among the person, environment, and behavior [20]. SCT further identifies self-efficacy, outcome expectations, barriers and facilitators, and self-regulation as core determinants of physical activity behavior. SCT asserts that positively impacting confidence in the ability to control health habits (self-efficacy), expected benefits of performing physical activity (outcome expectations), barriers and facilitators to engaging in physical activity, and self-management of goal setting and action planning may underpin change in physical activity behavior. Although behavior change interventions informed by SCT have been effective for increasing physical activity participation among colorectal cancer survivors [19], we are unaware of any SCT-grounded behavior change interventions for promoting physical activity specifically in adults with CD. The only study to investigate correlates of physical activity in CD identified a small, but significant association between exercise barriers and exercise benefits and self-reported physical activity [21].

We undertook a preliminary cross-sectional study that examined self-reported physical activity levels and SCT variables, including self-efficacy, outcome expectations, social support, and self-regulation, as correlates of physical activity behavior among adults with CD. We hypothesized that self-efficacy, outcome expectations, social support, and self-regulation would be positively correlated with physical activity levels. Our preliminary study may inform future research on modifiable correlates of physical activity and ultimately guide the development of behavior change interventions to increase physical activity and improve disease-related outcomes among adults with CD. 

## 2. Materials and Methods

This study received Institutional Review Board approval from the University of Alabama at Birmingham (UAB) and all participants provided informed consent before completing the survey. Participants were recruited from UAB Medicine electronic medical records and the UAB Digestive Health Clinic over a 7-month period. Study flyers were mailed to potentially eligible individuals or distributed by hand by the lead author (WNN). The flyers contained information about the study, inclusion criteria, as well as a website link and QR code to access the electronic survey. The inclusion criteria were (a) gastroenterologist confirmed diagnosis of Crohn’s disease and (b) between the ages of 18 and 65 as in similar studies examining physical activity in this population [10,22]. Interested participants accessed the informed consent and survey via the Qualtrics web platform between November 2022 and June 2023 by direct link or QR code. All participants who consented to the trial completed the survey within one week of opening the link. 

### 2.1. Electronic Survey

The electronic survey included a brief questionnaire assessing demographics and clinical characteristics (including age, sex, race, relationship status, education, income, disease duration, current medical therapy, and surgical history), the Harvey Bradshaw Index (HBI), the Godin Leisure Time Exercise Questionnaire (GLTEQ), the International Physical Activity Questionnaire-short form (IPAQ-SF), and 5 measures of SCT variables.

### 2.2. Disease Activity

The HBI incorporates several key aspects related to the severity of CD symptoms to assess clinical disease activity. This includes evaluating overall well-being, abdominal pain intensity, frequency of liquid stools, identification of any abdominal masses, and presence of complications associated with CD during the previous day [23]. The HBI utilizes a scoring system that ranges from 0–50, with scores ≥ 5 indicating active disease.

### 2.3. Physical Activity

This study used the GLTEQ and IPAQ-SF as self-report measures of physical activity. We opted for two self-report physical activity measures given the lack of consensus from researchers regarding the best measure to be used. Importantly, by assessing physical activity using the GLTEQ and IPAQ, we were able to compare physical activity scores from our study with median physical activity scores reported in other US studies examining physical activity behavior among adults with CD [10,22]. 

The GLTEQ assessed current physical activity level by measuring the frequency of strenuous, moderate, and light physical activity performed for periods of 15 min or more during the previous 7-day period [14]. We applied the most recent scoring method, known as the Health Contribution Score (HCS), to generate an overall GLTEQ score that aligns with current recommendations for physical activity and reflects the dose-response association between physical activity volume and health benefits [14]. The HCS focuses only on strenuous and moderate physical activities. It involves multiplying the frequencies of strenuous and moderate physical activity by 9 and 5 metabolic equivalents (METs), respectively, and then summing the weighted scores. The resulting HCS provides a continuous measure of exercise behavior ranging from 0 to 98. To further facilitate interpretation, the HCS was converted into a categorical variable with three levels: insufficiently active (<14 units), moderately active (14–23 units), or sufficiently active (≥24 units). 

The IPAQ-SF [15] offers a more focused assessment of physical activity by measuring frequency and duration of walking, vigorous physical activity, and moderate physical activity during the previous 7-day period. The short form of the IPAQ includes an additional question that measures time spent sitting during a typical weekday as an indicator of sedentary behavior. The IPAQ-SF weights each type of activity by its energy requirements to yield a score representing MET minutes/week. This consists of first multiplying the duration of vigorous, moderate, and walking activities by 8, 4, and 3.3 METs, respectively, and then again by the frequency (days per week) of each type of activity. The total weekly activity was then calculated by summing the scores for walking, vigorous-intensity, and moderate-intensity activity to form a continuous measure of physical activity in total MET minutes/week. The IPAQ-SF can yield categorical indicators of physical activity; however, there were missing values for duration of vigorous, moderate, and walking activities on the IPAQ-SF in almost half (*n* = 14) of the sample. As such, we utilized an established strategy [16] to calculate an IPAQ-SF metabolic equivalent that is similar with the GLTEQ HCS. The survey was first recoded by removing the duration of physical activity from the calculation of overall physical activity score. We then multiplied the frequency of vigorous, moderate, and walking activities by 8, 4, and 3.3, respectively, and summed the items as a continuous measure of physical activity. 

### 2.4. SCT Variables

The measures of SCT variables were chosen based on previously demonstrated test-retest reliability and validity in other chronic diseases (i.e., multiple sclerosis [24]). Self-efficacy for physical activity was assessed using a 6-item version of the Exercise Self-Efficacy (EXSE) Scale [25]. The EXSE assesses expectations regarding one’s confidence in sustaining regular physical activity over incremental, monthly periods. Participants rate items on the EXSE using a 100-point percentage scale, ranging from 0% (not at all confident) to 100% (highly confident), with responses recorded in 10-point increments. These items are averaged to generate a composite score, with higher values corresponding to increased levels of exercise self-efficacy.

Outcome expectations was measured using the 15-item Multidimensional Outcome Expectations for Exercise Scale (MOEES) [26]. The MOEES assesses individuals’ physical, social, and self-evaluative outcome expectations about physical activity. For the MOEES, participants rated how strongly they agree with each of the items on a scale ranging from 1 (Strongly disagree) to 5 (Strongly agree). The scores are summed into a total score with higher scores indicating more positive outcome expectations for exercise.

Social support was measured using the 24-item Social Provisions Scale (SPS) modified for physical activity [27]. The SPS assesses the extent individuals’ perceptions of social support influence physical activity behavior. Questions for the SPS are rated on a scale ranging from 1 (Strongly disagree) to 4 (Strongly agree). Items are summed for an overall score ranging from 24 to 96 with higher scores reflecting greater perceived social support for physical activity.

Exercise self-regulation was assessed using the Exercise Goal Setting Scale (EGS) and Exercise Planning and Scheduling Scale (EPS) [28]. The EGS includes items related to goal setting, self-monitoring, and problem solving, while the EPS consists of items related to incorporating exercise into one’s daily schedule. Each scale contains 10 items rated on a scale of 1 (Does not describe) through 5 (Completely describes). Items for both the EGS and EPS are summed into a total score with higher values indicating greater exercise goal setting and planning behaviors, respectively.

### 2.5. Analysis

All responses collected via Qualtrics were included in the data analysis, regardless of the amount of missing data on the surveys (i.e., cases with missing data were not removed and imputation was not performed for variables with missing data). As a result, the number of total responses for each survey varied. Data analysis was completed using SPSS version 29. Descriptive statistics are reported as mean ± standard deviation (SD) unless otherwise noted. We initially examined group differences (i.e., female vs. male, clinical remission vs. active disease) in demographic and clinical characteristics using independent samples *t*-tests (age, disease duration) or chi square (income, education, surgical history) analyses. The main analysis involved non-parametric, bivariate correlations (r_s_) based on possible risks of outliers and non-normality in a relatively small sample, and coefficients were interpreted such that 0.1, 0.3, and 0.5 were weak, moderate, and strong correlations, respectively [29]. Statistical significance was set a priori at *p* < 0.05 using a one-tailed test based on a hypothesized pattern of results.

## 3. Results

### 3.1. Descriptive and Clinical Characteristics

Descriptive information about the sample is reported in Table 1. The overall sample included 30 adults with CD (Males: 12; Females: 18) with a mean age of 37.4 (SD = 13.3) years and disease duration of 16.7 (SD = 11.1) years. Most of the sample (18/30, 60%) were in clinical remission as measured by the HBI (i.e., scores < 5), and more females reported prior surgery for CD than males [χ^2^ (1, *n* = 30) = 6.04), *p* = 0.02]. The sample included mostly White (27/30, 90%) participants with an annual income of $50,000 or more (23/30, 77%) and a post-secondary education degree (23/30, 77%).

### 3.2. Physical Activity Levels

Descriptive statistics for the physical activity and SCT variables in the overall sample are presented in Table 2. The overall GLTEQ score was 33.4 ± 19.4 (possible range 0–119 units) while the mean GTLEQ HCS was 24.4 ± 16 (possible range 0–98 units). When compared with the normative mean value of 45.8 reported for healthy adult participants by Godin and Shepard [26], the overall mean GLTEQ score in this study was significantly lower based on a one-sample *t*-test (*d* = 0.64; *p* < 0.01). Of note, 57% (17/30) of the sample was classified as active, 20% (6/30) as moderately active, and 23% (7/30) as insufficiently active per GLTEQ HCS scoring criteria. The median (IQR) total IPAQ-SF score was 1804.5 (769.9–2914.9) MET minutes/week for the 16 participants who reported both frequency and duration of walking, moderate, and vigorous activity. Regarding sedentary behavior, participants (*n* = 18) reported a median (IQR) sitting time of 450 (345–600) MET minutes/weekday. When compared with the normative median value of 240 MET minutes/weekday reported for healthy U.S. adults by Bauman et al. [30], the median sitting time for CD participants was significantly lower based on a one-sample median test (*p* < 0.001). The mean modified IPAQ-SF score (*n* = 28) was 39.1 ± 21.6, and there were weak to strong correlations among the physical activity measures: GLTEQ HCS and IPAQ-SF (r_s_ = −0.02); GLTEQ HCS and modified IPAQ-SF (r_s_ = 0.36), and IPAQ-SF and modified IPAQ-SF (r_s_ = 0.79). There were significant differences in physical activity levels by gender and surgical history—females (*n* = 18, *t* (28) = −2.55, *p* = 0.03) and participants who previously underwent surgery for CD (*n* = 18, *t* (28) = −2.17, *p* = 0.04) reported higher GLTEQ scores; no differences were identified based on scores from the modified IPAQ-SF. Physical activity levels based on GLTEQ HCS and modified IPAQ-SF scores did not differ by age, income, education, disease duration, or disease activity.

### 3.3. SCT Correlates of Physical Activity

The Spearman correlation matrix containing the major study variables is provided in Table 3. Participants reported high levels of exercise self-efficacy, social support, and outcome expectations, while mean values for the EPS and EGS indicated moderate exercise goal setting and planning behaviors. There were no statistically significant relationships between SCT variables and GLTEQ and total IPAQ-SF scores in the overall sample; however, a significant moderate positive correlation was noted for exercise goal setting and modified IPAQ-SF scores (r_s_ = 0.34, *p* = 0.05). Participants with greater exercise goal setting behaviors reported higher IPAQ-SF scores. Interestingly, sitting time as measured by the IPAQ-SF was indirectly associated with social support (r_s_ = −0.48, *p* = 0.02) and exercise goal setting (r_s_ = −0.49, *p* = 0.02), and the correlations approached the guideline for strong per Cohen [29].

There were significant correlations among scores from the SCT variables in the overall sample. Self-efficacy was directly and strongly associated with outcome expectations (r_s_ = 0.60, *p* = 0.001), social support (r_s_ = 0.55, *p* = 0.004), and planning (r_s_ = 0.62, *p* = 0.001), and moderately associated with goal setting (r_s_ = 0.40, *p* = 0.03), meaning participants with higher exercise self-efficacy reported more positive outcome expectations for exercise, higher perceived social support for exercise, and greater exercise goal setting and planning behaviors. Outcome expectations were directly and strongly related to goal setting (r_s_ = 0.68, *p* < 0.001) and moderately correlated with social support (r_s_ = 0.41, *p* = 0.04). Social support was directly related to goal setting (r_s_ = 0.63, *p* < 0.001) and planning (r_s_ = 0.58, *p* = 0.002), and there was a strong association between goal setting and planning (r_s_ = 0.51, *p* = 0.008).

## 4. Discussion

There is increasing evidence for physical activity behavior and its association with a diverse range of disease and health-related outcomes among adults with CD [9], yet overall low rates of physical activity participation. This supports the critical need for research into the development of effective behavior change interventions that promote uptake of physical activity in this population. To that end, examining physical activity behavior and its SCT correlates represents a meaningful first step in the design and implementation of behavioral interventions targeting physical activity for improving disease-related outcomes in persons with CD. Among the small sample of adults with CD, we observed that overall levels of physical activity were significantly lower than normative values for adults, but over half (57%) of participants reported high levels of physical activity and goal setting was correlated with physical activity levels. Although the results were derived from a smaller sample size, participants reported a median sitting time of 7.5 h/weekday, and sedentary time was associated with social support and goal setting. These findings may support the value of using a social cognitive framework for examining the factors associated with physical activity and sedentary behavior in CD. 

The mean GLTEQ score was significantly lower than the normative mean value for healthy adult participants reported by Godin and Shephard [26] (*t* = −3.5; *p* < 0.01); this difference in mean scores was moderate to large in magnitude (*d* = 0.64) and substantiates previous research reporting that adults with CD are significantly less physically active than non-diseased individuals [18,31,32]. Our data further indicated that 57% of participants engaged in levels of physical activity that would be classified as “sufficiently active” based on GLTEQ HCS criteria, yet median sitting time (450 min/day) was nearly twice as high as normative values for U.S. adults (240 min/day) [30] based on the IPAQ-SF. This is consistent with a cross-sectional study that utilized self-report and device-based measures of physical activity and similarly found that CD patients in remission spent more time lying down compared to healthy controls based on accelerometer data despite similar overall physical activity levels [33]. Our seemingly contradictory results regarding continuous and categorical GLTEQ HCS scores could be attributed to the bimodal distribution for the GLTEQ HCS, with similar proportions of participants categorized as sufficiently active (57%, GLTEQ HCS ≥ 24 units) and moderately to insufficiently active (43%, GLTEQ HCS < 24 units). This bimodal distribution suggests that although a majority may achieve adequate physical activity levels, a notable proportion of adults with CD might still struggle with engaging in regular physical activity. Collectively, these findings generally underscore the complexity of physical activity and sedentary behaviors in individuals with CD and emphasize the need for tailored interventions that promote sustained physical activity levels among this segment of the US population. 

We provide initial evidence that goal setting may be associated with physical activity among adults with CD. Results further suggest that goal setting and social support may be associated with sitting time. It is important to highlight that missing values for duration of sitting as well as vigorous, moderate, and walking activities on the IPAQ-SF were present in almost half (*n* = 14) of the sample; as such, the findings regarding the association between physical activity and goal setting are derived only from the modified version of the IPAQ-SF. Nonetheless, the observed correlation coefficient for the relationship between goal setting and physical activity (r_s_ = 0.34, *p* = 0.05) and sedentary behavior (r_s_ = −0.49, *p* = 0.02) implies notable associations [29], suggesting that goal setting may be worthy of focus in future interventions aimed at promoting physical activity and reducing sedentary behavior in individuals with CD. We further note that although self-efficacy was not significantly associated with physical activity, outcome expectations, social support, goal setting and planning were highly correlated with self-efficacy. This is important as self-efficacy (a person’s belief in their ability to perform a desired behavior [20]) is a focal determinant of physical activity behavior, impacting behavior both directly and through its influence on other determinants. Our findings generally align with previous studies involving healthy older adults [34], college-aged students [28], and persons with multiple sclerosis [24]. Those studies suggest that self-efficacy influences physical activity indirectly through goal setting, a self-regulatory strategy, and that goals have a moderate direct relationship with physical activity. Consequently, while the indirect relationship between self-efficacy and physical activity does not undermine the significance of self-efficacy as a factor, it supports the importance of considering other variables when exploring physical activity correlates in those with CD. To that end, further research among those with CD to examine these SCT-based constructs (i.e., self-efficacy, outcome expectations, social support, goal setting and planning) as modifiable factors within the exercise environment is warranted.

There are many strengths of this study, including (a) the focus on theory-based variables as correlates of physical activity; (b) the largest US study to directly examine self-reported physical activity levels and sedentary behavior; and (c) the use of validated measures for examining physical activity and sedentary behavior and its correlates in those with CD. However, this study is not without significant limitations. First, we recognize that the cross-sectional nature precludes inferences about causality. That is, in line with SCT’s foundation on reciprocal determinism [20], it is possible that physical activity impacts self-efficacy, outcome expectations, social support, and self-regulation rather than these variables impacting physical activity participation. In addition, based on the overall small sample size due to logistical restraints, it is possible our study was underpowered to detect a significant association between physical activity and SCT variables. Future studies that include a larger sample of adults with CD are necessary for accurate estimation of associations between SCT variables and physical activity. Lastly, our sample included a disproportionately high number of participants with post-secondary education degrees—as of 2019, approximately 35% of Americans held some type of post-secondary academic degree [35] whereas three-fourths of participants in our sample (77%) held a post-secondary education degree. Because research suggests individuals with advanced degrees are more likely to exercise regularly [36], it is possible that education level may have influenced regular exercise. Despite these limitations, we provide novel insight into variables that correlate with physical activity and could be targets for increasing physical activity participation among those with CD.

The understanding of exercise behaviors among adults with CD is expanding globally, and this study marks the first exploration of the correlation between physical activity and SCT variables in this population. As such, there are numerous avenues for future investigation into physical activity behavior among individuals with CD. One focus involves additional, theoretical-based research into factors influencing physical activity. Such studies should continue to utilize a social cognitive framework to explore physical activity behavior, while also considering other theories and frameworks for a more comprehensive understanding of physical activity behavior in CD. The Transtheoretical Model and Theory of Planned Behavior are two of the most commonly used theories in physical activity behavior change interventions among colorectal cancer survivors [19]. Another line of research involves addressing the possibility of measurement error (i.e., recall bias) when relying solely on self-reported physical activity by utilizing device-based measures. This will allow for a better understanding of daily physical activity patterns including sedentary behavior, light physical activity, and moderate-vigorous physical activity. Further, though limited data exists regarding enjoyment, exercise barriers, and exercise benefits among adults with CD [21,37,38,39], future research could examine specific barriers and facilitators of physical activity among those with CD.

## 5. Conclusions

Overall, we observed that over half (57%) of participants reported high levels of physical activity, yet the mean physical activity level was lower and median sitting time was higher than normative values in this small sample of adults with CD. We further provide initial evidence that goal setting may be directly correlated with physical activity levels, and although the sample size was smaller, goal setting and social support were indirectly associated with sedentary behavior. These findings support the value of using SCT for further examining the factors associated with physical activity behavior in CD. Future research should verify the associations between SCT constructs and physical activity behavior in a larger sample of adults with CD and then move forward with designing an intervention around the enhancement of self-efficacy and promotion of goal setting strategies for promotion of physical activity.

## Figures and Tables

**Table 1:** Demographic and clinical information for the overall sample of participants with Crohn’s disease.

Variable	Category	*n* (%)
**Sex**		
	Male	12 (40%)
	Female	18 (60%)
**Race**		
	White/Caucasian	27 (90%)
	Black/African American	2 (7%)
	Other	1 (3%)
**Relationship Status**		
	Married	16 (53%)
	Not married	14 (47%)
**Income**		
	<$50,000/year	7 (23%)
	≥$50,000/year	23 (77%)
**Education**		
	Completed college	23 (77%)
	Did not complete college	7(23%)
**HBI Score**		
	Clinical remission	18 (60%)
	Mild disease activity	5 (16.7%)
	Moderate disease activity	6 (20%)
	Severe disease activity	1 (3.3%)
**Current Medical Therapy**		
	Oral corticosteroid	0 (0%)
	5-ASAs	1 (4%)
	Anti-TNF Therapy	20 (83%)
	Immunomodulators	2 (8%)
Previous surgery *		18 (60%)

Note: *n* = 30. CD = Crohn’s disease; HBI = Harvey Bradshaw Index, HBI scores ranged 0–17 with a mean score of 4.4 (SD = 3.9). * Reflects the number and percentage of participants answering “yes” to this question.

**Table 2:** Descriptive information for physical activity and Social Cognitive Theory variables.

Variables	Category	*n*	
GLTEQ Total (mean ± SD (actual range))		30	33.4 ± 19.4 (0–91.0)
GLTEQ HCS (mean ± SD (actual range))		30	24.4 ± 16.2 (0–70.0)
	Active, n (%)	17	57%
	Moderately active, n (%)	6	20%
	Insufficiently active, n (%)	7	23%
IPAQ-SF (median (IQR))			
	Total physical activity (MET min/wk)	16	1804.5 (769.9–2914.9)
	Time spent sitting (MET min/day)	18	450 (345–600)
Modified IPAQ-SF (mean ± SD (actual range))		28	39.1 ± 21.6 (0–79.1)
SCT Variables (mean ± SD (actual range))			
	Exercise self-efficacy	26	70.9 ± 29.2 (8.3–100.0)
	Exercise outcome expectations	28	63.8 ± 6.0 (53.0–75.0)
	Exercise social support	27	70.6 ± 8.8 (53.0–90.0)
	Exercise goal setting	26	26.6 ± 9.0 (10.0–44.0)
	Exercise planning	26	25.4 ± 9.3 (12.0–43.0)

Note: GLTEQ = Godin Leisure-Time Exercise Questionnaire; GLTEQ HCS = Godin Leisure-Time Exercise Questionnaire Health Contribution Score; IPAQ-SF = short-form International Physical Activity Questionnaire; IQR = interquartile range; SCT = Social Cognitive Theory.

**Table 3:** Spearman correlation coefficients for the study variables for the overall sample of adults with Crohn’s disease.

Variable	1	2	3	4	5	6	7	8
**1** Physical activity (GLTEQ HCS)	1.00							
**2** Physical activity (modified IPAQ-SF)	0.36 *	1.00						
**3** Sedentary behaviors (IPAQ-SF)	0.38	0.36 *	1.00					
**4** Social support (SPS)	0.04	0.16	−0.48 *	1.00				
**5** Goal setting (EGS)	0.21	0.34 *	−0.49 *	0.63 **	1.00			
**6** Outcome expectations (MOEES)	0.04	0.04	−0.30	0.41 *	0.68 **	1.00		
**7** Self-efficacy (EXSE)	0.24	0.10	−0.09	0.55 **	0.40 *	0.60 **	1.00	
**8** Planning (EPS)	0.29	0.26	−0.27	0.58 **	0.51 **	0.33	0.62 **	1.00

Note: GLTEQ HCS = Godin Leisure-Time Exercise Questionnaire Health Contribution Score; IPAQ-SF = short-form International Physical Activity Questionnaire; SPS = Social Provisions Scale; EGS = Exercise Goal Setting Scale; MOEES = Multidimensional Outcome Expectations for Exercise Scale; EXSE = Exercise Self-Efficacy Scale; EPS = Exercise Planning Scale; * denotes statistical significance at *p* < 0.05; ** denotes statistical significance at *p* < 0.01.

## Data Availability

The data presented in this study are available on request from the corresponding author.

## References

[1] Shanahan F. (2002). Crohn’s disease. Lancet.

[2] Khrom M., Long M., Dube S., Robbins L., Botwin G.J., Yang S., Mengesha E., Li D., Naito T., Bonthala N.N. (2024). Comprehensive Association Analyses of Extraintestinal Manifestations in Inflammatory Bowel Disease. Gastroenterology.

[3] van der Have M., van der Aalst K.S., Kaptein A.A., Leenders M., Siersema P.D., Oldenburg B., Fidder H.H. (2014). Determinants of health-related quality of life in Crohn’s disease: A systematic review and meta-analysis. J. Crohn’s Colitis.

[4] Otto J.M., O’Doherty A.F., Hennis P.J., Mitchell K., Pate J.S., Cooper J.A., Grocott M.P.W., Montgomery H.E. (2012). Preoperative exercise capacity in adult inflammatory bowel disease sufferers, determined by cardiopulmonary exercise testing. Int. J. Color. Dis..

[5] Wiroth J.-B., Filippi J., Schneider S.M., Al-Jaouni R., Horvais N., Gavarry O., Bermon S., Hébuterne X. (2005). Muscle performance in patients with Crohn’s disease in clinical remission. Inflamm. Bowel Dis..

[6] Baban Y.N., Edicheria C.M., Joseph J., Kaur P., Mostafa J.A. (2021). Osteoporosis Complications in Crohn’s Disease Patients: Factors, Pathogenesis, and Treatment Outlines. Cureus.

[7] Van Langenberg D.R., Della Gatta P., Warmington S.A., Kidgell D.J., Gibson P.R., Russell A.P. (2014). Objectively measured muscle fatigue in Crohn’s disease: Correlation with self-reported fatigue and associated factors for clinical application. J. Crohn’s Colitis.

[8] Pekmezi D., Motl R. (2022). Targeting Physical Inactivity Using Behavioral Theory in Chronic, Disabling Diseases. Exerc. Sport Sci. Rev..

[9] Neal W.N., Jones C.D., Pekmezi D., Motl R.W. (2022). Physical Activity in Adults with Crohn’s Disease: A Scoping Review. Crohn’s Colitis 360.

[10] Jones P.D., Kappelman M.D., Martin C.F., Chen W., Sandler R.S., Long M.D. (2015). Exercise decreases risk of future active disease in patients with inflammatory bowel disease in remission. Inflamm. Bowel Dis..

[11] Robinson R.J., Krzywicki T., Almond L., Al F., Abrams K., Iqbal S.J., Mayberry J.F. (1998). Effect of a low-impact exercise program on bone mineral density in Crohn’s disease: A randomized controlled trial. Gastroenterology.

[12] Seeger W.A., Thieringer J., Esters P., Allmendinger B., Stein J., Schulze H., Dignass A. (2020). Moderate endurance and muscle training is beneficial and safe in patients with quiescent or mildly active Crohn’s disease. United Eur. Gastroenterol. J..

[13] Lee N., Radford-Smith G., Taaffe D.R. (2005). Bone loss in Crohn’s disease: Exercise as a potential countermeasure. Inflamm. Bowel Dis..

[14] Godin G. (2011). The Godin-Shephard Leisure-Time Physical Activity Questionnaire. Health Fit. J. Can..

[15] Craig C.L., Marshall A.L., Sjöström M., Bauman A.E., Booth M.L., Ainsworth B.E., Pratt M., Ekelund U.L., Yngve A., Sallis J.F. (2003). International physical activity questionnaire: 12-country reliability and validity. Med. Sci. Sports Exerc..

[16] Gosney J.L., Scott J.A., Snook E.M., Motl R.W. (2007). Physical activity and multiple sclerosis: Validity of self-report and objective measures. Fam. Community Health.

[17] Gatt K., Schembri J., Katsanos K.H., Christodoulou D., Karmiris K., Kopylov U., Pontas C., Koutroubakis I.E., Foteinogiannopoulou K., Fabian A. (2019). Inflammatory Bowel Disease [IBD] and Physical Activity: A Study on the Impact of Diagnosis on the Level of Exercise Amongst Patients with IBD. J. Crohn’s Colitis.

[18] Van Langenberg D.R., Papandony M.C., Gibson P.R. (2015). Sleep and physical activity measured by accelerometry in Crohn’s disease. Aliment. Pharmacol. Ther..

[19] Mbous Y.P., Patel J., Kelly K.M. (2020). A systematic review and meta-analysis of physical activity interventions among colorectal cancer survivors. Transl. Behav. Med..

[20] Bandura A. (2004). Health promotion by social cognitive means. Health Educ. Behav..

[21] Tew G.A., Jones K., Mikocka-Walus A. (2016). Physical Activity Habits, Limitations, and Predictors in People with Inflammatory Bowel Disease: A Large Cross-sectional Online Survey. Inflamm. Bowel Dis..

[22] Crumbock S.C., Loeb S.J., Fick D.M. (2009). Physical activity, stress, disease activity, and quality of life in adults with Crohn disease. Gastroenterol. Nurs..

[23] Harvey R.F., Bradshaw J.M. (1980). A simple index of Crohn’s-disease activity. Lancet.

[24] Suh Y., Weikert M., Dlugonski D., Sandroff B., Motl R.W. (2011). Social cognitive correlates of physical activity: Findings from a cross-sectional study of adults with relapsing-remitting multiple sclerosis. J. Phys. Act. Health.

[25] McAuley E. (1993). Self-efficacy and the maintenance of exercise participation in older adults. J. Behav. Med..

[26] Wójcicki T.R., White S.M., McAuley E. (2009). Assessing outcome expectations in older adults: The multidimensional outcome expectations for exercise scale. J. Gerontol. Ser. B Psychol. Sci. Soc. Sci..

[27] Cutrona C.E., Russell D.W. (1987). The provisions of social relationships and adaptation to stress. Adv. Pers. Relatsh..

[28] Rovniak L.S., Anderson E.S., Winett R.A., Stephens R.S. (2002). Social cognitive determinants of physical activity in young adults: A prospective structural equation analysis. Ann. Behav. Med..

[29] Cohen J. (1988). Statistical Power Analysis for the Behavioral Sciences.

[30] Bauman A., Ainsworth B.E., Sallis J.F., Hagströmer M., Craig C.L., Bull F.C., Pratt M., Venugopal K., Chau J., Sjöström M. (2011). The descriptive epidemiology of sitting. A 20-country comparison using the International Physical Activity Questionnaire (IPAQ). Am. J. Prev. Med..

[31] Mack D.E., Wilson P.M., Gilmore J.C., Gunnell K.E. (2011). Leisure-time physical activity in Canadians living with Crohn disease and ulcerative colitis: Population-based estimates. Gastroenterol. Nurs. Off. J. Soc. Gastroenterol. Nurses Assoc..

[32] Qalqili T.R., Rayyan Y.M., Tayyem R.F. (2021). Lifestyle and dietary factors associated with inflammatory bowel disease among jordanian patients. J. Gastrointest. Liver Dis..

[33] Cabalzar A.L., de Azevedo F.M., de Azevedo Lucca F., de Moura Reboredo M., Malaguti C., Chebli J.M.F. (2019). Physical activity in daily life, exercise capacity, and quality of life in patients with Crohn’s disease on inflximad-induced remission: A preliminary study. Arq. Gastroenterol..

[34] Anderson E.S., Wojcik J.R., Winett R.A., Williams D.M. (2006). Social-cognitive determinants of physical activity: The influence of social support, self-efficacy, outcome expectations, and self-regulation among participants in a church-based health promotion study. Health Psychol..

[35] Creech W.L., Towner B.C., Battista R.A. (2022). Physical Activity among Adults in Rural Western North Carolina during the COVID-19 Pandemic. Prev. Chronic Dis..

[36] Zajacova A., Lawrence E. (2021). Postsecondary Educational Attainment and Health among Younger U.S. Adults in the “College-for-All” Era. Socius.

[37] Tew G.A., Leighton D., Carpenter R., Anderson S., Langmead L., Ramage J., Faulkner J., Coleman E., Fairhurst C., Seed M. (2019). High-intensity interval training and moderate-intensity continuous training in adults with Crohn’s disease: A pilot randomised controlled trial. BMC Gastroenterol..

[38] Bottoms L., Leighton D., Carpenter R., Anderson S., Langmead L., Ramage J., Faulkner J., Coleman E., Fairhurst C., Seed M. (2019). Affective and enjoyment responses to 12 weeks of high intensity interval training and moderate continuous training in adults with Crohn’s disease. PLoS ONE.

[39] Jones K., Naisby J., Baker K., Tew G.A. (2023). Exercise Perceptions and Experiences in Adults with Crohn’s Disease Following a Combined Impact and Resistance Training Program: A Qualitative Study. Crohn’s Colitis 360.

